# Transarterial Microembolization in Tendinopathies: From Experimental to Evidence-Based Therapy

**DOI:** 10.1007/s00270-025-04184-7

**Published:** 2025-09-09

**Authors:** P. Minko, A. Taheri Amin

**Affiliations:** 1 Department of Diagnostic and Interventional Radiology, Medical Faculty, University Duesseldorf, Moorenstrasse 5, 40225 Duesseldorf, Germany; 2 Department of Diagnostic and Interventional Radiology, Charité Universitätsmedizin Berlin, Corporate Member of Freie Universität Berlin and Humboldt-Universität Zu Berlin, Berlin, Germany

Chronic tendinopathies remain challenging to treat, especially when conservative measures fail. Transcatheter arterial microembolization (TAME) has emerged as a treatment alternative for tendinopathies, with its efficacy demonstrated in a meta-analysis across various indications [1].

Sugihara et al. presented the first multicenter retrospective analysis evaluating TAME for patients with chronic achilles tendinopathy (AT) refractory to conservative management [2]. Eighty-two patients from four institutions underwent TAME with imipenem/cilastatin. Hypervascularity was assessed using Doppler ultrasound and digital subtraction angiography (DSA), with the introduction of a novel angiographic grading system ranging from Grade 0 (no blush) to Grade 3 (massive blush with early venous return). Over 24 months, pain scores (Numeric rating scale; NRS) improved from 6.7 to 1.5, Victorian Institute of Sports Assessment (VISA-A) scores from 48.4 to 82.2, and clinical success— ≥ 50% NRS reduction at 12 months—was achieved in 81.7% of patients.

Until now, evidence of TAME for AT has been confined to cohorts with short-term follow-up [1]. This work provides the first long-term data in a large cohort of patients with AT, demonstrating not only durable clinical benefit but also indicating superiority over established non-surgical options. With a mean age of 47 years, the study population was considerably younger than in other TAME studies, such as those on knee osteoarthritis. Notably, even within the subgroup of competitive athletes—who have the highest functional demands—more than half of the patients achieved clinical success. These findings further support the positioning of TAME as a first-line therapy for chronic pain even in young and active individuals seeking full functional recovery.

Another major strength of the study is the detailed technical description. The DSA-based blush classification proposed is an important step toward standardizing musculoskeletal embolization and could be applied to other TAME procedures to enable reporting of currently heterogeneous procedural parameters. As higher blush grades in this cohort were associated with greater clinical success, the classification also carries immediate therapeutic relevance, further enhancing its value.

The authors report that in some cases, superselective catheterization was not possible, resulting in reflux into the lower foot arteries. Combined with the tendon’s sparse, non-redundant arterial supply, this increases the risk of non-target embolization and supports the use of temporary embolic agents when treating end-artery territories such as in AT. In contrast, rich anastomotic networks in genicular artery embolization require treatment of multiple branches, with larger volumes of even permanent embolics [3, 4].

Since the introduction of temporary embolics, the question has arisen whether superselective catheterization is truly necessary or if the technically simpler and less radiation-intensive unselective delivery from the main feeding artery is sufficient. Sugihara et al. demonstrated that clinical success rates were significantly higher after superselective than selective embolization. Thus, while temporary embolics enhance the safety profile of TAME, the optimal catheter position and embolization endpoint vary across different indications, fueling a shift in the technical discourse away from a one-size-fits-all model toward patient- and disease-tailored selection of embolic agents and embolization technique.

The drawback of the study is its retrospective design. The cohort was heterogeneous regarding symptom chronicity and prior treatments, which may affect generalizability. Imaging was not performed routinely pre- and post-intervention, limiting reproducibility and external validity of the blush grading system.

In summary, this study presents the largest cohort and longest follow-up to date for TAME in AT, providing robust long-term efficacy data. Prospective randomized trials are needed to refine technical standards, validate imaging endpoints, and define patient- and disease-specific parameters for tailored strategies. Looking ahead, TAME could evolve from a novel alternative to a precision-targeted, evidence-based therapy, ultimately reshaping the landscape of interventional pain medicine.
